# Vaccination strategies for measles control and elimination: time to strengthen local initiatives

**DOI:** 10.1186/s12916-020-01843-z

**Published:** 2021-01-05

**Authors:** F. T. Cutts, M. J. Ferrari, L. K. Krause, A. J. Tatem, J. F. Mosser

**Affiliations:** 1 Department of Infectious Disease Epidemiology, London School of Hygiene and Tropical Medicine, London, UK; 2 Center for Infectious Disease Dynamics, Pennsylvania State University, University Park, PA USA; 3 Vaccine Delivery, Global Development, The Bill & Melinda Gates Foundation, Seattle, WA USA; 4 WorldPop, Department of Geography and Environmental Science, University of Southampton, Highfield, Southampton, SO17 1BJ UK; 5 Institute for Health Metrics and Evaluation, University of Washington, Seattle, WA 98121 USA

**Keywords:** Measles, Routine immunization, Campaigns, Elimination

## Abstract

**Background:**

Through a combination of strong routine immunization (RI), strategic supplemental immunization activities (SIA) and robust surveillance, numerous countries have been able to approach or achieve measles elimination. The fragility of these achievements has been shown, however, by the resurgence of measles since 2016. We describe trends in routine measles vaccine coverage at national and district level, SIA performance and demographic changes in the three regions with the highest measles burden.

**Findings:**

WHO-UNICEF estimates of immunization coverage show that global coverage of the first dose of measles vaccine has stabilized at 85% from 2015 to 19. In 2000, 17 countries in the WHO African and Eastern Mediterranean regions had measles vaccine coverage below 50%, and although all increased coverage by 2019, at a median of 60%, it remained far below levels needed for elimination. Geospatial estimates show many low coverage districts across Africa and much of the Eastern Mediterranean and southeast Asian regions. A large proportion of children unvaccinated for MCV live in conflict-affected areas with remote rural areas and some urban areas also at risk. Countries with low RI coverage use SIAs frequently, yet the ideal timing and target age range for SIAs vary within countries, and the impact of SIAs has often been mitigated by delays or disruptions. SIAs have not been sufficient to achieve or sustain measles elimination in the countries with weakest routine systems. Demographic changes also affect measles transmission, and their variation between and within countries should be incorporated into strategic planning.

**Conclusions:**

Rebuilding services after the COVID-19 pandemic provides a need and an opportunity to increase community engagement in planning and monitoring services. A broader suite of interventions is needed beyond SIAs. Improved methods for tracking coverage at the individual and community level are needed together with enhanced surveillance. Decision-making needs to be decentralized to develop locally-driven, sustainable strategies for measles control and elimination.

## Background

The measles vaccine has prevented more child deaths than any other vaccine in use today. In 1994, building upon the momentum created by expanded routine immunization (RI) coverage and progress towards polio elimination, the region of the Americas established a goal to eliminate measles. In 2016, the strong political commitment to elimination led to the region achieving this goal, through a combination of ‘keeping up’ high RI coverage, catch-up supplementary immunization activities (SIAs) up to age 15 years to fill immunity gaps among cohorts missed by RI in earlier years and interrupt measles transmission, occasional follow-up SIAs up to age 5 years and close monitoring of coverage and disease surveillance with swift action to respond to outbreaks [1]. ‘Speed-up’ SIAs up to 30 or 39 years of age, conducted primarily for rubella elimination [2], probably also contributed.

The rapid fall in measles incidence in the Americas after the catch-up campaigns encouraged other regions to adopt these strategies, initially to pursue goals of measles mortality reduction and subsequently measles elimination. The level of measles population immunity required to sustain measles elimination (the so-called herd immunity threshold) is generally estimated to be above 90% (discussed in Cutts et al. (2020) [3]); hence, the World Health Organization (WHO) recommends that countries aiming at measles elimination should achieve ≥ 95% coverage with both doses equitably to all children in every district [4].

Despite dramatic reductions in global reported incidence and estimated mortality, success has not been uniform [5, 6]. Countries that have not fully implemented and sustained these strategies have never interrupted transmission and have experienced some of the largest recorded outbreaks [5]. Others had initial success but did not implement keep-up and follow-up strategies well enough to avoid major resurgences after long periods of low incidence [6, 7]. Globally, from a nadir of 132,490 in 2016, reported cases increased each year to 869,770 in 2019, the highest number since 1996 [8], with over half a million cases reported from just two countries—the Democratic Republic of the Congo (DRC) and Madagascar [9].

This resurgence underscores the tenuous status of current global and regional measles control efforts—put even further at risk by immunization disruptions caused by the COVID-19 pandemic [10]. In this paper, we review trends in the main drivers of measles burden—RI coverage, SIA performance and demographic changes—in the WHO African (AFR), Eastern Mediterranean (EMR) and southeast Asian (SEAR) regions, which have the highest estimated measles mortality, and propose a change in priorities for measles control strategies post-COVID-19.

### Trends in routine measles immunization

WHO-UNICEF national estimates of immunization coverage (WUENIC) show that global coverage of the first dose of measles-containing vaccine (MCV1) coverage soared from 16% in 1980 to 68% in 1989, rose slightly to 71% in 1999, increased to 83% by 2009, and then stabilized at 85% from 2015 to 19. All 17 countries in AFR and EMR with WUENIC MCV1 < 50% in 2000 had increased coverage by 2019 to a median of 60%, but their coverage remained far below levels needed for elimination. Furthermore, coverage remained very low in 2019 in Angola (51%), Cameroon (60%), Central African Republic (49%), Chad (41%), DRC (57%), Ethiopia (58%), Guinea (47%), Somalia (46%), South Sudan (49%) and Nigeria (54%) [11]. A second dose of MCV (MCV2) was rarely part of routine schedules in countries eligible for support from the GAVI Vaccine Alliance (GAVI) until 2010 since when introductions have accelerated with 60% WUENIC MCV2 achieved by 2019.

**Fig. 1:**
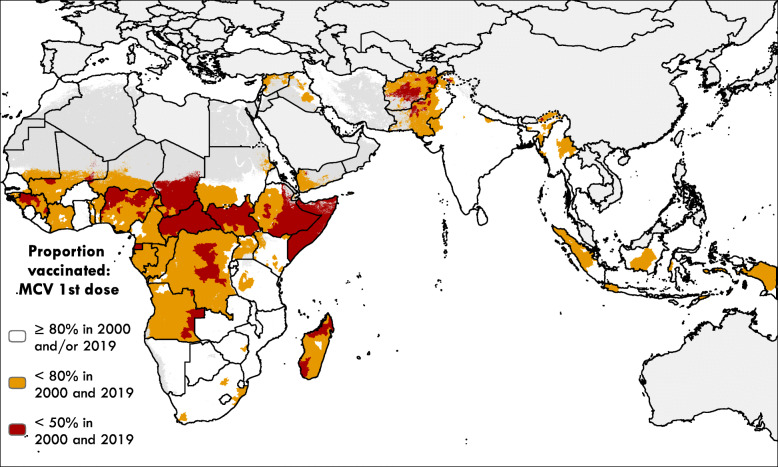
Areas of low MCV1 coverage in both 2000 and 2019 in AFR, EMR and SEAR. MCV1 coverage at the second administrative level (district or equivalent units) was estimated by Sbarra et al. [12] using survey data and geostatistical models. Areas classified as ‘barren or sparsely vegetated’ based on European Space Agency Climate Change Initiative (ESA-CCI) satellite data [15] or with fewer people than 10 per 1 × 1-km pixel based on WorldPop estimates [16] are masked in dark grey. Countries not included in the Sbarra et al. analysis are masked in light grey

Geospatial analyses of survey data have allowed estimation of subnational and local patterns of MCV1 coverage [12–14] and show that coverage can vary substantially within countries and over time. Figure 1 shows the results of geospatial analyses of survey data from 2000 to 2019; details of the methods are described elsewhere [12]. Although MCV1 coverage in most districts and countries increased from 2000 to 2019, there is a band of districts across AFR from Nigeria to Somalia, additional large areas of Guinea and Angola and parts of Afghanistan and Pakistan where coverage was estimated to be below 50% in 2000 and still below 50% in 2019 [12]. MCV1 coverage remained below 80% in both 2000 and 2019 at the district level in much of AFR, EMR and parts of most countries in SEARO. By contrast, few districts in the Americas had estimated MCV1 coverage persistently below 80% in both 2000 and 2019.

**Fig. 2:**
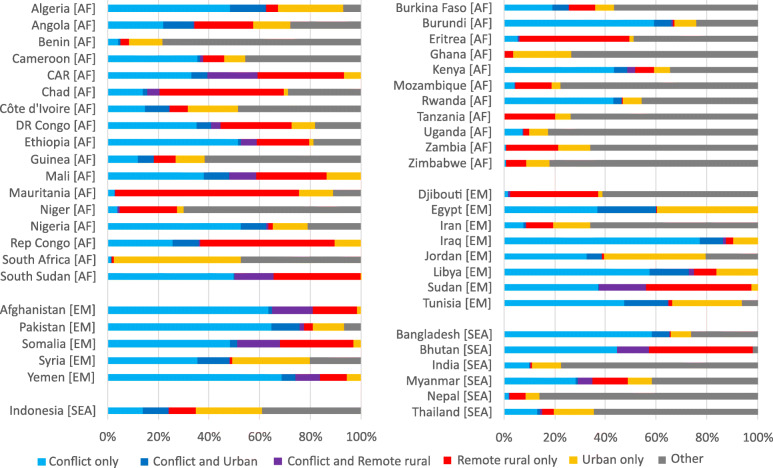
Plots showing the estimated breakdown of under-1-year-olds not receiving MCV by characteristic per country for countries with a full set of data, in AFR, EMR and SEAR. Panel **a** (left) shows countries with MCV1 coverage ≤ 80% and panel **b** (right) shows countries with MCV1 coverage > 80% according to WUENIC 2018. Estimated numbers of unvaccinated children were calculated from geospatial estimates of MCV1 coverage using methods described in Sbarra et al. [12] and population estimates from WorldPop [16], using the geographic distribution of < 1-year-old children in each country as a proxy for the geographic distribution of children of the target age for MCV1 vaccination. Conflict areas were identified using data from the ACLED [17] and Uppsala [18] conflict data programmes, 2018. The programmes provide geolocated data on conflict events, and here (following Wagner et al. [19]), conflicts resulting in fatalities in the 2 years prior to the period of study were aggregated and a buffer of 50 km was applied to the conflict fatalities data to identify ‘conflict-affected’ areas. Remote areas were defined as those with travel time > 3 h to the nearest settlement of > 50,000 people using estimates from Weiss et al. [20] and the distribution and extent of urban areas were identified using estimates from the Global Human Settlement framework [21]

Clustering of unvaccinated individuals poses risks for local disease outbreaks but could also facilitate targeted interventions. Known contributors to spatial inequity include remoteness, conflict and urban slums. For countries with available data, Fig. 2 shows the estimated geospatial distribution of children of the target age who did not receive MCV1 in 2017, in relation to conflict-affected, urban and remote rural areas. Direct comparison between countries is limited by potential differences in the completeness of data on conflict, but it is clear that most unvaccinated children in EMR live in conflict-affected areas, as do those in some of the largest African countries such as DRC, Ethiopia and Nigeria. A high proportion of unvaccinated children live in remote rural locations in Chad, DRC, Ethiopia, Mauritania and the Republic of Congo (Fig. 2a). Overall, a relatively small proportion (~ 10%) of unvaccinated children lived in urban areas, but analyses to date have not distinguished the urban poor from other urban populations. Of note, none of these factors is identified for over half the unvaccinated children in many of the higher coverage countries (Fig. 2b). Other socio-economic and cultural factors have recently been found to be consistently associated with higher vaccination uptake around the globe, including high confidence in vaccines, high trust in health care workers, higher levels of science education, younger age and high information-seeking behaviour, while in some countries, belonging to a minority religious group or refusing to state religious belief was associated with lower uptake [22]. Even when mothers are willing to accept vaccination, coverage may be low due to health systems barriers such as inadequate communications about vaccination, unreliability of sessions and high transport costs to reach them [23]. These analyses suggest that current RI programmes often produce unequal levels of RI throughout a country and fail to reach children in high-risk populations. Of note, during the elimination program in the Americas, high-risk groups were identified using coverage and surveillance data and targeted efforts developed to reach them, with cross-border collaboration where needed [2].

For countries to reduce reliance on SIAs, routine services must be capable of providing high and equitable coverage with MCV1 and MCV2. Where this is not the case, measles continues to cause major morbidity and mortality. From 2013 to 2018, the highest incidence rates occurred in low- and middle-income countries (LMICs), especially those that had low or zero historical coverage of MCV2, an indicator of RI strength [6]. Highest incidence rates were in unvaccinated pre-schoolers in each region, although incidence was also high in older persons in some LMICS such as Madagascar and in high-income countries, where outbreaks followed many years of low or absent transmission.

### SIAs

From 2000 to 2019, AFR reported the vaccination of 1.3 billion children via SIAs, EMR 700 million and SEAR 750 million [24]. According to data from 81 post-campaign coverage surveys (PCCS) reported to WHO from these regions, 56 (69%) of SIAs reached at least 90% of the target population [24]. Although SIAs can attain higher and more equitable coverage than RI [13, 25, 26], SIA coverage in countries with weaker health systems has rarely approached the levels that would be needed for elimination [26, 27]. Furthermore, the extent to which SIAs reach children missed by RI is a key determinant of impact but has only recently begun to be evaluated. PCCS are encouraged to report these data but surveys often exclude conflict-affected areas and ascertainment of prior vaccination status is of unknown accuracy [26].

To reduce transmission, SIAs need to increase population immunity. Because SIAs target many individuals who will already have been eligible for RI or had measles infection, the effective increase in population immunity is much lower than the nominal coverage of SIAs. Estimates of SIA impact on population immunity provide more complete information. Trentini et al. [28] incorporated data from sero-surveys in dynamic transmission models to estimate that SIAs generated about 45% of the immunized fraction of the population in Ethiopia and about 25% in Kenya (which had higher RI coverage) in 2015. Thakkar et al. [29] used measles surveillance data in their models to estimate that SIAs conducted between 2012 and 2017 in Pakistan immunized 40% of the susceptible individuals reached by the campaigns. In China, similar analyses estimated that provincial-level SIAs became more effective over time. Those conducted from 1996 to 2005 resulted in estimates of 0.5% to 45% reductions in susceptible individuals in the target age classes while SIAs from 2006 to 11 resulted in 32% to 87% reductions [30]. These useful estimates, however, are available for very few LMICS.

High-quality SIAs can help to eliminate measles, but to maintain elimination—unless routine coverage of MCV1 and MCV2 is extremely high—SIAs must be repeated frequently enough to maintain the susceptible population below the herd-immunity threshold. Predicting when to conduct follow-up SIAs is challenging. As vaccination programmes improve, measles incidence varies more from year to year, highlighted by recent outbreaks in Madagascar, Mongolia and others [6]. This increase in inter-annual variation is predicted by theoretical models of the epidemic dynamics as countries approach the elimination threshold [31]. Periodic additional interventions, such as SIAs and outbreak response vaccination, may reduce mean incidence over many years but lead to larger outbreaks in any 1 year in settings where RI is not sufficient to prevent the rapid increase of susceptible cohorts following campaigns [32, 33]. This increased variation in periodicity can make it harder to decide when to conduct follow-up SIAs.

The optimum timing of SIAs often also varies within a country. The minimally sufficient SIA interval depends on both the birth rate and the RI coverage, which govern the decrease in population immunity in between campaigns [33, 34]. Sub-national variation in birth rates and vaccination coverage mean that SIA intervals based on national rates may be either insufficient or more frequent than necessary to maintain elimination in any sub-national unit [33, 35].

To compound difficulties in choosing the desired interval, delays to planned SIAs are not uncommon, e.g. due to delays in obtaining funding and logistical support, political changes, natural or man-made disasters [36, 37]. Appropriate timing of SIAs is critical to immunize susceptible individuals before an increase in transmission occurs [29]. Major outbreaks have been reported shortly before a scheduled follow-up SIA in e.g. Burkina Faso, DRC and Kenya [36, 37] while delays in implementing outbreak response vaccination campaigns may allow widespread transmission to occur [38].

### Demographic changes

Along with immunization coverage, demographic changes can drive trends in measles incidence. Merler et al. [39] and Li et al. [30] illustrated that measles incidence declined faster than expected by vaccination coverage alone due to concomitant reductions in birth rates in Italy and China, respectively. Where birth rates are high, RI coverage must be higher [40] and SIAs must be more frequent [34] to maintain a given level of population immunity. From 1980 to 2018, crude birth rates per 1000 population fell much less in AFR than in other regions (from 46.7 to 34.9 in AFR, 42.4 to 25.7 in EMR and 35.6 to 17.6 in SEAR). From 2000 to 2018, the population aged under 15 years increased by over 50% in AFR, by about 24% in EMR and remained stable in SEAR. At given RI and SIA coverage, this increase means a greater density of unvaccinated children and higher risk of measles transmission.

Regional and national demographic summaries may mask significant subnational variations in risk profiles. For example, high rates of rural to urban migration have seen urban areas grow more rapidly than rural areas, with differing demographic profiles [41]. The influx of susceptible persons, from areas with lower access to vaccination but also low measles transmission, to crowded urban areas facilitates measles transmission [42, 43], especially if recent migrants are not recognized officially and vaccinated in a timely fashion. Cyclical rural-urban migration, as illustrated in Niger [44] and Pakistan [29], may further limit access to RI and affect the performance of SIAs. Improved socio-demographic data need to be incorporated into strategic planning of measles elimination.

## Conclusions

Despite tremendous progress in reducing measles burden worldwide through immunization and demographic changes over the last four decades, the global measles situation in 2020 remains tenuous. Increased measles incidence and outbreaks in many LMICS result from stagnant or falling and unequal RI coverage and suboptimal SIA implementation [6]. In this fragile moment, the COVID-19 pandemic is worsening immunization vulnerabilities, putting the world at even higher risk for major outbreaks of measles and other vaccine-preventable diseases [45].

The postponement of SIAs due to the COVID-19 pandemic provides an opportunity to increase the priority given to RI and to implement a range of strategies to enhance its reach. To overcome fears and rumours relating to COVID-19, substantial community engagement will be required to plan, promote and monitor services. This could provide a foundation for building stronger services post-COVID. At health facilities and outreach sites, increased attention and funding is needed to implement strategies to diagnose and remedy causes of missed opportunities for vaccination [46] and other barriers to utilization of services such as stockouts and cancellation of sessions due to lack of transport or human resources [47]. The COVID-19 pandemic has led to a re-emphasis on administering MCV (and other missed vaccination doses) to eligible children older than 12 months [48], and this message needs reinforcement after the pandemic to enhance routine MCV1 and MCV2 coverage. In urban areas, vaccination sessions should be conducted more frequently with flexible, extended hours to facilitate attendance by working mothers. Vaccination services should be designed in collaboration with target communities including urban slums and interventions designed to vaccinate recent arrivals in, and visitors to, cities [49]. Systems for tracking the vaccination status of every child, with active follow-up of those behind on routine vaccinations need to be strengthened [50]. Routine services should aim to fill immunity gaps beyond infancy.

Some of the lowest coverage countries have conducted national SIAs at 2–3 yearly intervals since 2001, such that SIAs have become a strategy to compensate for weak RI rather than interrupt transmission. Other approaches could be more effective and efficient. For example, conducting regular national Periodic Intensification of Routine Immunization (PIRI) could be easier to plan with shorter lead time than SIAs, for which funding applications are required 12–18 months in advance and delays are common. This would also mean that children missed through other strategies receive MCV at a younger age than if they wait 2–3 years for the next SIA, thus increasing the chance of protecting the child before exposure to measles. Subnational approaches also need evaluation to tailor activities to the local demographic and security situations. The experience of microplanning gained in SIAs needs to be translated into improved planning and intensification of RI, including better planning of outreach sites or mobile team use according to geospatial data and more rational human resources deployment that maximizes the use of community-based workers. New approaches to estimating population numbers at fine spatial scales through the use of satellite building mapping, surveys and geostatistical models [51] should provide better estimates of population denominators than the use of simple projections from census baselines that can be decades old. The collection of recent enumeration data from small sample areas, or the use of listing data from recent surveys, can provide training data for statistical models that utilize relationships between these enumeration data and geospatial covariates to estimate population numbers in unsampled areas, together with uncertainty metrics. Examples of application of such approaches have been shown recently for Nigeria [52], Zambia [53] and DRC [54], with outputs available to explore at https://apps.worldpop.org/woprVision. Better information on population denominators, demographics and mobility will improve planning and monitoring.

A large proportion of children unvaccinated for MCV live in remote rural and conflict-affected areas. In these places, flexible approaches developed at the local level such as multiple rounds of vaccination using mobile teams [55] can be used during lulls in fighting [56, 57]. Resources need to be decentralized to facilitate district-level decision-making and rapid action when access to previously difficult areas is possible. When people flee conflict-affected areas, vaccination needs to be provided at the first opportunity at their destinations. Strong community-based communication and support mechanisms will enable this [57].

In the Americas, close monitoring of coverage and disease surveillance were priority components of the elimination plan and led to timely action when gaps were identified. These systems need revitalizing in LMICs. Prioritizing the implementation of data quality improvement plans should improve the accuracy of routinely reported data on doses administered via RI and SIAs by age group, along with program inputs. Digitizing information can help programmes track each child’s vaccination status and improve coverage monitoring [50]. Measles surveillance, which is currently extremely insensitive, needs substantial investment to improve the investigation of suspect cases and root cause analysis of outbreaks. Laboratory confirmation is greatly constrained by the need to transport specimens to central laboratories; hence, the roll-out of point of care diagnostics [58] should help increase the specificity of reporting in more remote areas. As routine coverage improves and the interval between measles outbreaks lengthens, there is a risk of unrecognized accumulation of susceptible persons in older ages [6]; hence, it is important to use multiple sources of data to identify population immunity gaps.

Countries and their international partners face difficult decisions for maintaining health services during COVID-19 and revitalizing them afterwards. There will probably be unprecedented demands for national campaigns aiming to catch-up quickly for service disruption, but reinvigorating RI is essential in areas which have so far failed to attain high RI coverage. SIAs have an important role in reducing measles transmission and filling immunity gaps but are currently not sufficient to achieve or sustain measles control in the weakest countries. We believe that this is a time to reflect on methods to strengthen health systems, including routine surveillance and immunization, in the countries and districts where RI coverage has remained low for two decades or more. A broader range of strategies needs evaluation to ensure that RI improves while reducing inequities in a sustainable way [59], and create conditions that would make future measles elimination feasible. Now, more than ever, political will is needed to fund the required structural changes in immunization programmes to protect all persons against measles and other vaccine-preventable diseases [60].

## Data Availability

The data used for Fig. 1 are accepted for publication by Nature and all of the estimates used to construct the figure will be available on http://ghdx.healthdata.org/lbd-data at the time of publication. The data used for constructing Fig. 2 are all openly available: Age structured population: www.worldpop.org; Remoteness: https://www.nature.com/articles/nature25181; Conflict: https://ucdp.uu.se/; https://acleddata.com/#/dashboard; Urban/rural: https://ghsl.jrc.ec.europa.eu/ghs_smod2019.php
